# Supplementary material for: Chemical Constituents and Anticancer Activities of Marine-Derived Fungus Trichoderma lixii
Source: Molecules. 2024 Apr 29;29(9):2048. doi: 10.3390/molecules29092048 (PMC11085134; doi:10.3390/molecules29092048)
Supplement: Supplementary file 1 [file molecules-29-02048-s001.zip › molecules-2921839-supplementary.pdf]

Supplementary Materials

Chemical constituents and anticancer activities of marine-derived fungus *Trichoderma lixii*

Natchanun Sirimangkalakitti [1], Jianyu Lin [1], Kazuo Harada [1], Andi Setiawan [2], Mitsuhiro Arisawa [1,*] and Masayoshi Arai [1,*]

[1] Graduate School of Pharmaceutical Sciences, Osaka University, 1-6 Yamadaoka, Suita, Osaka 565-0871, Japan; siriman@phs.osaka-u.ac.jp (N.S.), lin-j@phs.osaka-u.ac.jp (J.L), harada6@phs.osaka-u.ac.jp (K.H.)
[2] Department of Chemistry, Faculty of Science, Lampung University, Jl. Dr. Sumantri Brodjonegoro No. 1, Bandar Lampung 35145, Indonesia; andi.setiawan@fmipa.unila.ac.id (A.S.)
* Correspondence: arisaw@phs.osaka-u.ac.jp (Mi.A.); araim@phs.osaka-u.ac.jp (Ma.A.)

List of supporting information

Physical and spectral data of trichodermamide A (**1**)

Physical and spectral data of trichodermamide B (**2**)

Physical and spectral data of aspergillazine A (**3**)

Physical and spectral data of DC1149B (**4**)

Physical and spectral data of ergosterol peroxide (**5**)

Physical and spectral data of cerebrosides D/C (**6**/**7**) and their proposed MS cleavage pathway

Physical and spectral data of 5-hydroxy-2,3-dimethyl-7-methoxychromone (**8**)

Physical and spectral data of nafuredin A (**9**)

Physical and spectral data of harzianumols E/F (**10**/**11**)

Physical and spectral data of trichodermamide A (**1**)[1]

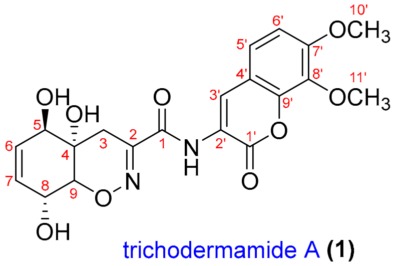

trichodermamide A (**1**)

1.0 mg
Pale yellow amorphous solid
$[\alpha]_D^{26}$ +59° (c 0.10, MeOH); lit. $[\alpha]_D^{15}$ +128° (c 0.15, MeOH)
UV λ_{max} 203, 250, 332 nm; lit. UV λ_{max} 250, 334 nm
HR-MALDI-MS *m/z* 455.1064 $[M+Na]^+$, calcd for $C_{20}H_{20}N_2O_9Na$, 455.1061

Table S1. ^1H NMR (600 MHz) and ^{13}C NMR (150 MHz) data for trichodermamide A (**1**) in DMSO-d_6

position	δ_H (Multiplicity)	δ_C (Type)
NH	9.34 (1H, s)	
1		161.0 (C)
2		150.2 (C)
3	2.48 (1H, d, *J* = 20.1 Hz)<br>2.04 (1H, d, *J* = 20.1 Hz)	23.1 (CH_2)
4		67.4 (C)
5	4.23 (1H, d, *J* = 3.0 Hz)	73.2 (CH)
6	5.45 (1H, d, *J* = 10.5 Hz)	129.9 (CH)
7	5.40 (1H, d, *J* = 10.5 Hz)	128.1 (CH)
8	4.00 (2H, s)	66.2 (CH)
9		83.9 (CH)
4-OH	5.28 (1H, s)	
5-OH	5.32 (1H, d, *J* = 5.4 Hz)	
8-OH	5.45 (1H, s)	
1'		157.9 (C)
2'		121.0 (C)
3'	8.55 (1H, s)	123.7 (CH)
4'		113.7 (C)
5'	7.52 (1H, d, *J* = 8.7 Hz)	123.1 (CH)
6'	7.15 (1H, d, *J* = 8.7 Hz)	110.1 (CH)
7'		153.8 (C)
8'		135.2 (C)
9'		143.6 (C)
OCH_3-10'	3.90 (3H, s)	56.4 (CH_3)
OCH_3-11'	3.84 (3H, s)	60.9 (CH_3)

[1] Garo, E.; Starks, C.M.; Jensen, P.R.; Fenical, W.; Lobkovsky, E.; Clardy, J. Trichodermamides A and B, Cytotoxic Modified Dipeptides from the Marine-Derived Fungus *Trichoderma virens*. *J Nat Prod* **2003**, 66, 423-426.

Physical and spectral data of trichodermamide B (**2**)[1]

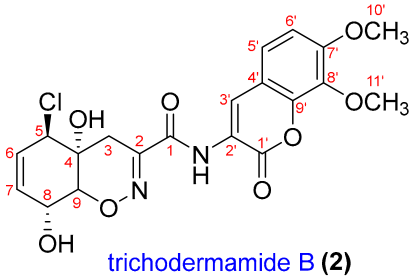

trichodermamide B **(2)**

3.0 mg
Colorless amorphous powder
$[\alpha]_D^{26}$ +103° (c 0.16, MeOH); lit. $[\alpha]_D^{15}$ +111° (c 0.15, MeOH)
UV λ_{max} 203, 252, 345 nm; lit. UV λ_{max} 252, 344 nm
HR-MALDI-MS *m/z* 473.0722 $[M+Na]^+$, calcd for $C_{20}H_{19}N_2O_8Na^{35}Cl$, 473.0722

Table S2. ^{1}H NMR (600 MHz) and ^{13}C NMR (150 MHz) data for trichodermamide B (**2**) in $CDCl_3$+DMSO-d_6

position	δ_H (Multiplicity)	δ_C (Type)
NH	9.35 (1H, s)	
1		160.9 (C)
2		149.8 (C)
3	2.75 (1H, dd, *J* = 19.2, 1.8 Hz)<br>2.15 (1H, d, *J* = 19.2 Hz)	25.2 (CH_2)
4		67.5 (C)
5	4.77 (1H, br s)	64.9 (CH)
6	5.52 (2H, m)	129.3 (CH)
7		127.5 (CH)
8	4.03 (1H, m)	65.8 (CH)
9	4.19 (1H, dd, *J* = 7.8, 1.8 Hz)	84.1 (CH)
4-OH	5.42 (1H, s)	
8-OH	5.29 (1H, d, *J* = 5.4 Hz)	
1'		158.0 (C)
2'		120.9 (C)
3'	8.50 (1H, s)	124.1 (CH)
4'		113.9 (C)
5'	7.11 (1H, d, *J* = 8.7 Hz)	122.5 (CH)
6'	6.82 (1H, d, *J* = 8.7 Hz)	109.3 (CH)
7'		154.0 (C)
8'		135.8 (C)
9'		143.9 (C)
OCH_3-10'	3.85 (3H, s)	61.3 (CH_3)
OCH_3-11'	3.83 (3H, s)	56.3 (CH_3)

Physical and spectral data of aspergillazine A (**3**)[2,3]

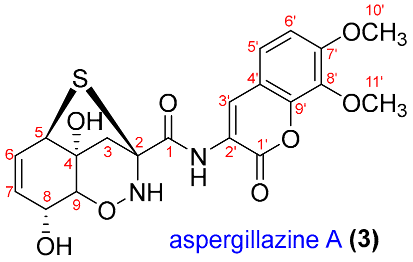

aspergillazine A **(3)**

9.6 mg
Pale yellow powder
$[\alpha]_D^{26}$ -273° (c 0.08, MeOH); lit. $[\alpha]_D^{20}$ -356° (c 0.14, MeOH)[3]
UV λ_{max} 205, 244, 337 nm; lit. UV λ_{max} 205, 245, 337 nm[2]
HR-MALDI-MS *m/z* 471.0834 $[M+Na]^+$, calcd for $C_{20}H_{20}N_2O_8NaS$, 471.0833

Table S3. ^1H NMR (600 MHz) and ^{13}C NMR (150 MHz) data for aspergillazine A (**3**) in CD_3OD[2]

position	δ_H (Multiplicity)	δ_C (Type)
1		162.1 (C)
2		77.2 (C)
3	3.12 (1H, d, *J* = 11.7 Hz)<br>2.39 (1H, d, *J* = 11.7 Hz)	50.8 (CH_2)
4		76.1 (C)
5	4.14 (1H, d, *J* = 5.0 Hz)	47.7 (CH)
6	5.93 (1H, dd, *J* = 9.9, 5.0 Hz)	126.1 (CH)
7	6.06 (1H, dd, *J* = 9.9, 5.1 Hz)	128.4 (CH)
8	4.34 (1H, br d, *J* = 3.6 Hz)	65.2 (CH)
9	4.20 (1H, br s)	81.8 (CH)
1'		158.0 (C)
2'		121.2 (C)
3'	6.97 (1H, s)	118.4 (CH)
4'		115.3 (C)
5'	7.04 (1H, d, *J* = 8.4 Hz)	129.4 (CH)
6'	6.64 (1H, d, *J* = 8.4 Hz)	105.8 (CH)
7'		155.9 (C)
8'		137.9 (C)
9'		148.9 (C)
OCH_3-10'	3.88 (3H, s)	56.4 (CH_3)
OCH_3-11'	3.81 (3H, s)	61.2 (CH_3)

[2] Capon, R.J.; Ratnayake, R.; Stewart, M.; Lacey, E.; Tennant, S.; Gill, J.H. Aspergillazines A-E: novel heterocyclic dipeptides from an Australian strain of *Aspergillus unilateralis*. *Org Biomol Chem* **2005**, 3, 123-129.
[3] Yamazaki, H.; Rotinsulu, H.; Takahashi, O.; Kirikoshi, R.; Namikoshi, M. Induced production of a new dipeptide with a disulfide bridge by long-term fermentation of marine-derived *Trichoderma* cf. *brevicompactum*. *Tetrahedron Lett* **2016**, 57, 5764-5767.

Physical and spectral data of DC1149B (**4**)[4]

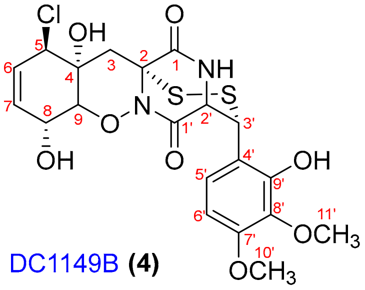

DC1149B **(4)**

11.0 mg
Pale yellow powder
$[\alpha]_D^{25}$ -206° (c 0.04, MeOH); lit. $[\alpha]_D^{20}$ -288° (c 0.10, MeOH)
UV λ_{max} 204, 280, 342 nm; lit. UV λ_{max} 207, 280 nm
HR-MALDI-MS *m/z* 539.0319 $[M+Na]^+$, calcd for $C_{20}H_{21}N_2O_8NaS_2{}^{35}Cl$, 509.0320

Table S4. ^1H NMR (600 MHz) and ^{13}C NMR (150 MHz) data for DC1149B (**4**) in DMSO-d_6

position	δ_H (Multiplicity)	δ_C (Type)
NH	9.06 (1H, br s)	
1		166.5 (C)
2		69.9 (C)
3	2.13 (1H, d, *J* = 15.3 Hz)<br>2.00 (1H, d, *J* = 15.3 Hz)	33.0 (CH_2)
4		69.9 (C)
5	4.81 (1H, s)	67.0 (CH)
6	5.52 (1H, d, *J* = 10.2 Hz)	126.4 (CH)
7	5.58 (1H, d, *J* = 10.2 Hz)	131.1 (CH)
8	4.30 (1H, br s)	64.0 (CH)
9	4.00 (1H, d, *J* = 6.6 Hz)	85.4 (CH)
4-OH	5.62 (1H, br s)	
8-OH	5.36 (1H, br s)	
1'		164.3 (C)
2'	4.39 (1H, br s)	58.7 (CH)
3'	4.45 (1H, s)	44.8 (CH)
4'		116.2 (C)
5'	7.37 (1H, d, *J* = 9.0 Hz)	122.8 (CH)
6'	6.49 (1H, d, *J* = 9.0 Hz)	103.2 (CH)
7'		152.8 (C)
8'		135.7 (C)
9'		147.7 (C)
9'-OH	9.38 (1H, s)	
OCH_3-10'	3.73 (3H, s)	55.6 (CH_3)
OCH_3-11'	3.62 (3H, s)	60.1 (CH_3)

[4] Yamazaki, H.; Takahashi, O.; Murakami, K.; Namikoshi, M. Induced production of a new unprecedented epitrithiodiketopiperazine, chlorotrithiobrevamide, by a culture of the marine-derived *Trichoderma* cf. *brevicompactum* with dimethyl sulfoxide. *Tetrahedron Lett* **2015**, 56, 6262-6265.

Physical and spectral data of ergosterol peroxide (**5**)[5,6]

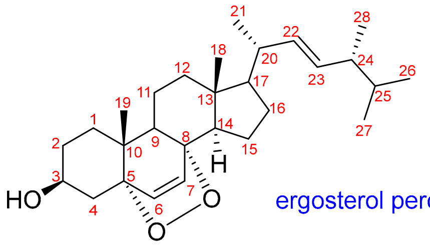

ergosterol peroxide **(5)**

0.9 mg
Colorless solid
$[\alpha]_D^{22}$ -18° (c 0.08, $CHCl_3$); lit. $[\alpha]_D^{25}$ -26° (c 0.2, $CHCl_3$)[6]
HR-MALDI-MS *m/z* 451.3183 $[M+Na]^+$, calcd for $C_{28}H_{44}O_3Na$, 451.3188

Table S5. ^{1}H NMR (600 MHz) and ^{13}C NMR (150 MHz) data for ergosterol peroxide (**5**) in $CDCl_3$[5,6]

position	δ_H (Multiplicity)	δ_C (Type)
1	1.95 (1H, m)<br>1.69 (1H, dt, *J* = 13.8, 3.6 Hz)	34.8 (CH_2)
2	1.85 (1H, m)<br>1.56 (1H, m)	30.2 (CH_2)
3	3.97 (1H, m)	66.6 (CH)
4	2.12 (1H, ddd, *J* = 13.7, 4.8, 1.5 Hz)<br>1.93 (1H, d, *J* = 13.7 Hz)	37.0 (CH_2)
5		82.3 (C)
6	6.25 (1H, d, *J* = 8.4 Hz)	135.5 (CH)
7	6.51 (1H, d, *J* = 8.4 Hz)	130.9 (CH)
8		79.6 (C)
9	1.50 (1H, m)	51.2 (CH)
10		37.1 (C)
11	1.51 (2H, m)	23.5 (CH_2)
12	1.95 (1H, m)<br>1.23 (1H, m)	39.4 (CH_2)
13		44.7 (C)
14	1.58 (1H, m)	51.8 (CH)
15	1.61 (2H, m)	20.8 (CH_2)
16	1.75 (1H, m)<br>1.35 (1H, m)	28.8 (CH_2)
17	1.22 (1H, m)	56.3 (CH)
18	0.81 (3H, s)	13.0 (CH_3)
19	0.88 (3H, s)	18.3 (CH_3)
20	2.01 (1H, m)	39.9 (CH)
21	1.00 (3H, d, *J* = 6.6 Hz)	21.0 (CH_3)
22	5.14 (1H, dd, *J* = 15.2, 8.7 Hz)	135.3 (CH)
23	5.22 (1H, dd, *J* = 15.2, 7.8 Hz)	132.4 (CH)
24	1.85 (1H, m)	42.9 (CH)
25	1.46 (1H, m)	33.2 (CH)
26, 27	0.81 (3H, d, *J* = 6.6 Hz)<br>0.83 (3H, d, *J* = 6.6 Hz)	19.8 (CH_3)<br>20.1 (CH_3)
28	0.90 (3H, d, *J* = 6.6 Hz)	17.7 (CH_3)

---

[5] Kim, D.S.; Baek, N.I.; Oh, S.R.; Jung, K.Y.; Lee, I.S.; Kim, J.H.; Lee, H.K. Anticomplementary activity of ergosterol peroxide from *Naematoloma fasciculare* and reassignment of NMR data. *Arch Pharm Res* **1997**, 20, 201-205.
[6] Lee, I.S.; Kim, J.P.; Na, M.K.; Jung, H.J.; Min, B.S.; Bae, K.H. Cytotoxicity of ergosterol derivatives from the fruiting bodies of *Hygrophorus russula*. *Nat Prod Sci* **2011**, 17, 85-89.

Physical and spectral data of cerebrosides D/C (**6**/**7**)[7]

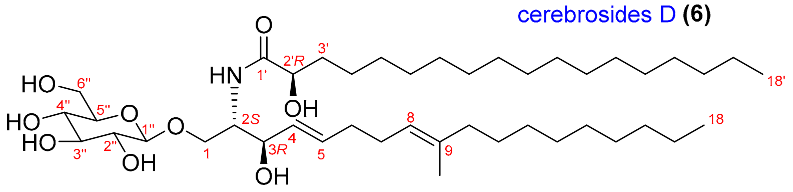

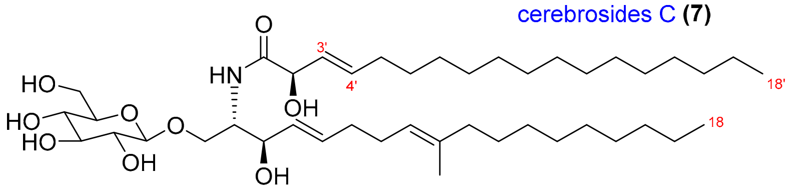

1.3 mg, mixture of cerebrosides **6** (major) and **7** (minor)
Colorless solid
$[\alpha]_D^{18}$ -2° (c 0.07, MeOH); lit. **6** $[\alpha]_D^{25}$ +8° (c 0.25, MeOH); **7** $[\alpha]_D^{23}$ -9° (c 0.23, MeOH)
UV λ_{max} 203 nm
HR-MALDI-MS *m/z* 778.5797 $[M+Na]^+$, calcd for $C_{43}H_{81}NO_9Na$, 778.5809 (**6**); *m/z* 776.5641 $[M+Na]^+$, calcd for $C_{43}H_{79}NO_9Na$, 776.5653 (**7**)

The length of the sphingoid long-chain base (LCB, C1-C18) and amide-linked long-chain fatty acid base (C1'-C18') were suggested as 18 carbons based on positive LC-MS/MS fragment ions[10]
6 *m/z* 756.7 $[M+H]^+$, 738.3 $[M+H-H_2O]^+$, 576.2 $[M+H-Glu]^+$, 558.5 $[M+H-Glu-H_2O]^+$, 396.6 $[M+H-Glu-LCB]^+$, 378.2 $[M+H-Glu-LCB-H_2O]^+$
7 *m/z* 754.7 $[M+H]^+$, 736.3 $[M+H-H_2O]^+$, 574.3 $[M+H-Glu]^+$, 556.6 $[M+H-Glu-H_2O]^+$, 394.6 $[M+H-Glu-LCB]^+$, 376.4 $[M+H-Glu-LCB-H_2O]^+$

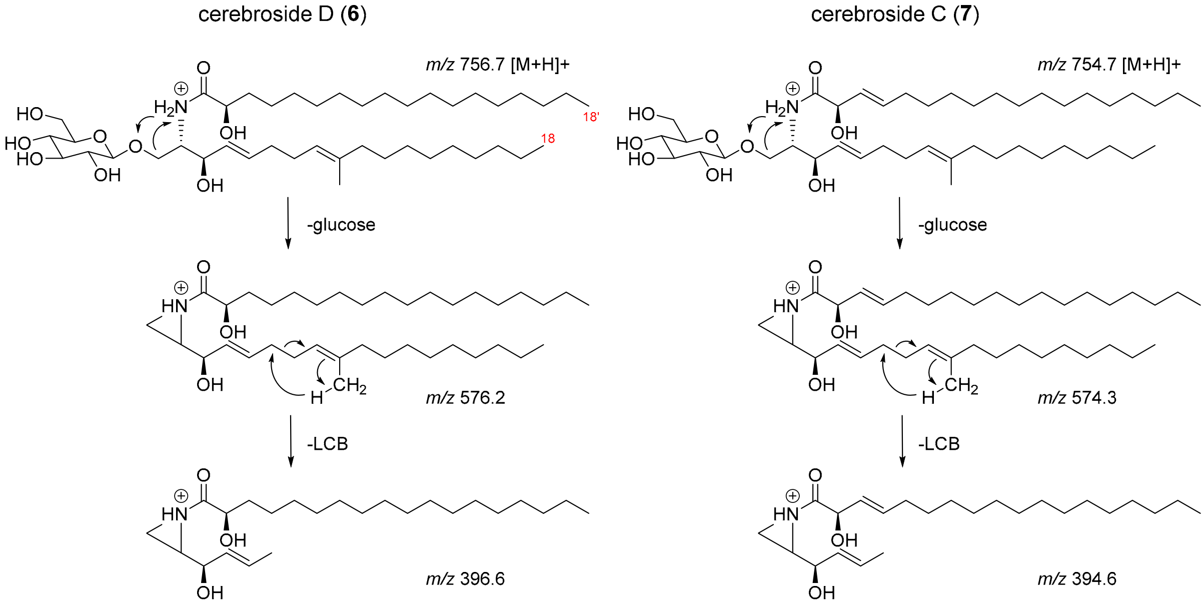

Figure S1. Proposed MS cleavage pathway of cerebrosides D (**6**) and C (**7**)

[7] Jiang, T.; Li, T.; Li, J.; Fu, H.Z.; Pei, Y.H.; Lin, W.H. Cerebroside analogues from marine-derived fungus *Aspergillus flavipes*. *J Asian Nat Prod Res* **2004**, 6, 249-257.

Table S6. ^{1}H NMR (600 MHz) and ^{13}C NMR (150 MHz) data for cerebrosides D/C (**6**/**7**)

position	**6** in DMSO-d_6 [7]		**6** in CD_3OD		**7** in CD_3OD	
	δ_H (Multiplicity)	δ_C (Type)	δ_H (Multiplicity)	δ_C (Type)	δ_H (Multiplicity)	δ_C (Type)
1	3.93 (1H, m)<br>3.50 (1H, m)	68.8 (CH_2)	4.12 (1H, m)<br>3.71 (1H, dd, *J* = 10.5, 3.3 Hz)	69.74 (CH_2)	4.12 (1H, m)<br>3.71 (1H, dd, *J* = 10.5, 3.3 Hz)	69.65 (CH_2)
2	3.80 (1H, m)	52.9 (CH)	3.985 (1H, m)	54.58 (CH)	3.985 (1H, m)	54.61 (CH)
3	3.98 (1H, m),	70.5 (CH)	4.14 (1H, m)	72.9 (CH)	4.14 (1H, m)	72.9 (CH)
4	5.40 (1H, dd, *J* = 15.0, 6.6 Hz)	131.09 (CH)	5.48 (1H, dd, *J* = 15.0, 7.2 Hz)	131.1 (CH)	5.49 (1H, m)	131.1 (CH)
5	5.54 (1H, m)	131.07 (CH)	5.74 (1H, dt, *J* = 15.0, 6.0 Hz)	134.67 (CH)	5.72 (1H, m)	134.5 (CH)/<br>134.74 (CH)
6	1.94 (2H, m)	32.2 (CH_2)	2.05 (2H, m)	33.8 (CH_2)	2.05 (2H, m)	33.8 (CH_2)
7	1.97 (2H, m)	27.4 (CH_2)	2.07 (2H, m)	28.7 (CH_2)	2.07 (2H, m)	28.8 (CH_2)
8	5.09 (1H, t, *J* = 6.0 Hz)	123.5 (CH)	5.15 (1H, td, *J* = 6.8, 0.9 Hz)	124.8 (CH)	5.15 (1H, td, *J* = 6.8, 0.9 Hz)	124.9 (CH)
9		135.0 (C)		136.8 (C)		136.7 (C)
10	1.91 (2H, t, *J* = 7.5 Hz)	39.5 (CH_2)	1.98 (2H, t, *J* = 7.5 Hz)	40.8 (CH_2)	1.98 (2H, t, *J* = 7.5 Hz)	40.8 (CH_2)
11				29.12 (CH_2)		29.14 (CH_2)
12-15	1.23 (br s)	28.7-32.2 (CH_2)	1.29 (br s)<br>1.39 (m)<br>1.41 (m)	30.2-30.9 (CH_2)	1.29 (br s)<br>1.39 (m)	30.2-30.9 (CH_2)
16				33.1 (CH_2)		33.1 (CH_2)
17	1.25 (overlapped)	22.2 (CH_2)	1.31 (overlapped)	23.8 (CH_2)	1.31 (overlapped)	23.8 (CH_2)
18	0.85 (3H, t, *J* = 6.9 Hz)	14.0 (CH_3)	0.90 (3H, t, *J* = 6.9 Hz)	14.5 (CH_3)	0.90 (3H, t, *J* = 6.9 Hz)	14.5 (CH_3)
9-CH_3	1.54 (3H, s)	15.8 (CH_3)	1.60 (3H, s)	16.1 (CH_3)	1.60 (3H, s)	16.1 (CH_3)
NH	7.41 (1H, d, *J* = 9.0 Hz)					
1'		173.8 (C)		177.2 (C)		177.2 (C)
2'	3.80 (1H, m)	71.0 (CH)	3.991 (1H, m)	73.1 (CH)	4.44 (1H, d, *J* = 7.2 Hz)	74.1 (CH)
3'				33.1 (CH_2)	5.49 (1H, m)	129.0 (CH_2)
4'			1.29 (br s)<br>1.39 (m)<br>1.41 (m)	26.2 (CH_2)	5.83 (1H, m)	134.5 (CH)/<br>134.74 (CH)
5'	1.23 (br s)	28.7-32.2 (CH_2)		30.2-30.9 (CH_2)	2.04 (2H, m)	33.4 (CH_2)
6'-15'					1.29 (br s)<br>1.39 (m)	30.2-30.9 (CH_2)
16'				33.1 (CH_2)		33.1 (CH_2)
17'	1.25 (overlapped)	22.2 (CH_2)	1.31 (overlapped)	23.8 (CH_2)	1.31 (overlapped)	23.8 (CH_2)
18'	0.85 (3H, t, *J* = 6.9 Hz)	14.0 (CH_3)	0.90 (3H, t, *J* = 6.9 Hz)	14.5 (CH_3)	0.90 (3H, t, *J* = 6.9 Hz)	14.5 (CH_3)
1''	4.11 (1H, d, *J* = 7.8 Hz)	103.6 (CH)	4.270 (1H, d, *J* = 8.0 Hz)	104.7 (CH)	4.273 (1H, d, *J* = 7.8 Hz)	104.7 (CH)
2''	2.94 (1H, m)	73.4 (CH)	3.19 (1H, dd, *J* = 9.3, 8.0 Hz)	75.0 (CH)	3.20 (1H, dd, *J* = 9.3, 7.8 Hz)	75.0 (CH)
3''	3.08 (1H, m)	76.9 (CH)	3.35 (1H, m)	77.9 (CH)	3.35 (1H, m)	77.9 (CH)
4''	3.03 (1H, m)	70.0 (CH)	3.28 (1H, m)	71.6 (CH)	3.28 (1H, m)	71.6 (CH)
5''	3.12 (1H, m)	76.6 (CH)	3.27 (1H, m)	78.0 (CH)	3.27 (1H, m)	78.0 (CH)
6''	3.66 (1H, m)<br>3.42 (1H, m)	61.1 (CH_2)	3.87 (1H, dd, *J* = 11.7, 1.8 Hz)<br>3.67 (1H, dd, *J* = 11.7, 4.8 Hz)	62.7 (CH_2)	3.87 (1H, dd, *J* = 11.7, 1.8 Hz)<br>3.67 (1H, dd, *J* = 11.7, 4.8 Hz)	62.7 (CH_2)
3-OH	4.93 (1H, d, *J* = 5.4 Hz)					
2'-OH	4.95 (1H, d, *J* = 4.2 Hz)					
2''-OH	4.96 (1H, d, *J* = 5.4 Hz)					
3''-OH	4.99 (1H, d, *J* = 4.2 Hz)					
4''-OH	5.00 (1H, d, *J* = 4.2 Hz)					
6''-OH	4.54 (1H, t, *J* = 5.7 Hz)					

Physical and spectral data of 5-hydroxy-2,3-dimethyl-7-methoxychromone (**8**)[8]

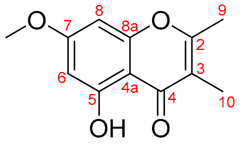

5-hydroxy-2,3-dimethyl-
7-methoxychromone **(8)**

0.8 mg
Colorless solid
UV λ_{max} 243, 290 nm; lit. UV λ_{max} 204, 245, 255sh, 289, 314 nm
HR-MALDI-MS *m/z* 221.0809 $[M+H]^+$, calcd for $C_{12}H_{13}O_4$, 221.0808

Table S7. ^1H NMR (600 MHz) and ^{13}C NMR (150 MHz) data for 5-hydroxy-2,3-dimethyl-7-methoxychromone (**8**) in $CDCl_3$

position	δ_H (Multiplicity)	δ_C (Type)
2		162.6 (C)
3		115.3 (C)
4		182.1 (C)
4a		104.8 (C)
5		162.2 (C)
6	6.31 (1H, s)	97.8 (CH)
7		165.2 (C)
8	6.31 (1H, s)	91.9 (CH)
8a		157.7 (C)
9	2.38 (3H, s)	18.6 (CH_3)
10	2.00 (3H, s)	9.3 (CH_3)
5-OH	12.94 (1H, s)	
7-OCH_3	3.84 (3H, s)	55.8 (CH_3)

[8] Takenaka, Y.; Tanahashi, T.; Nagakura, N. 2, 3-Dialkylchromones from mycobiont cultures of the lichen *Graphis scripta*. *Heterocycles* **2000**, 53, 1589-1593.

Physical and spectral data of nafuredin A (**9**)[9,10]

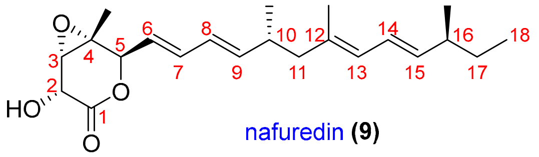

nafuredin **(9)**

5.1 mg
White solid
$[\alpha]_D^{21}$ +50° (c 0.50, $CHCl_3$); lit. $[\alpha]_D^{25}$ +49° (c 0.10, $CHCl_3$)[10]
UV λ_{max} 245 nm; lit. UV λ_{max} 253 nm[9,10]
HR-ESI-MS *m/z* 361.2364 $[M+H]^+$, calcd for $C_{22}H_{33}O_4$, 361.2379

Table S8. ^1H NMR (600 MHz) and ^{13}C NMR (150 MHz) data for 5-hydroxy-2,3-dimethyl-7-methoxychromone (**8**) in $CDCl_3$[9]

position	δ_H (Multiplicity)	δ_C (Type)
1		170.7 (C)
2	4.58 (1H, s)	68.2 (CH)
3	3.52 (1H, s)	58.7 (CH)
4		58.4 (C)
5	4.94 (1H, d, *J* = 8.0 Hz)	80.3 (CH)
6	5.49 (1H, dd, *J* = 15.3, 8.0 Hz)	122.1 (CH)
7	6.37 (1H, dd, *J* = 15.3, 10.5 Hz)	138.1 (CH)
8	6.01 (1H, dd, *J* = 15.5, 10.5 Hz)	126.2 (CH)
9	5.80 (1H, dd, *J* = 15.5, 7.2 Hz)	145.3 (CH)
10	2.43 (1H, m)	35.0 (CH)
11	2.09 (1H, dd, *J* = 13.5, 7.2 Hz)<br>1.98 (1H, dd, *J* = 13.5, 7.8 Hz)	47.3 (CH_2)
12		133.8 (C)
13	5.76 (1H, d, *J* = 11.0 Hz)	127.1 (CH)
14	6.17 (1H, dd, *J* = 15.2, 11.0 Hz)	124.7 (CH)
15	5.46 (1H, dd, *J* = 15.2, 7.8 Hz)	139.0 (CH)
16	2.07 (1H, m)	38.8 (CH)
17	1.32 (2H, quint, *J* = 7.2 Hz)	30.0 (CH_2)
18	0.86 (3H, t, *J* = 7.2 Hz)	11.9 (CH_3)
4-CH_3	1.47 (3H, s)	17.9 (CH_3)
10-CH_3	0.97 (3H, d, *J* = 6.6 Hz)	19.6 (CH_3)
12-CH_3	1.70 (3H, s)	16.6 (CH_3)
16-CH_3	0.99 (3H, d, *J* = 6.6 Hz)	20.3 (CH_3)

[9] Ui, H.; Shiomi, K.; Yamaguchi, Y.; Masuma, R.; Nagamitsu, T.; Takano, D.; Sunazuka, T.; Namikoshi, M.; Omura, S. Nafuredin, a novel inhibitor of NADH-fumarate reductase, produced by *Aspergillus niger* FT-0554. *J Antibiot (Tokyo)* **2001**, 54, 234-238.
[10] Damour, H.; Okoye, F.; Proksch, P.; Hakiki, A.; Mosaddak, M.; Hegazy, M.; Debbab, A. Pretrichodermamide A and nafuredin from *Trichoderma* sp, an endophyte of *Cola nitida*. *J Mater Environ Sci* **2015**, 6, 779-783.

Physical and spectral data of harzianumols E/F (**10/11**)[11]

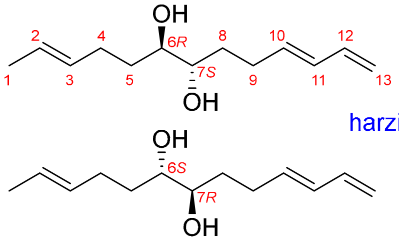

harzianumols E/F **(10/11)**

2.0 mg, mixture of enantiomers
Colorless solid
$[\alpha]_D^{19}$ -2° (c 0.20, $CHCl_3$)
UV λ_{max} 225 nm
HR-MALDI-MS *m/z* 233.1515 $[M+Na]^+$, calcd for $C_{13}H_{22}O_2Na$, 233.1517

Table S9. ^1H NMR (600 MHz) and ^{13}C NMR (150 MHz) data for harzianumols E/F (**10/11**) in DMSO-d_6

position	δ_H (Multiplicity)	δ_C (Type)
1	1.60 (3H, d, *J* = 4.8 Hz)	17.9 (CH_3)
2	5.38 (1H, m)	124.0 (CH)
3	5.41 (1H, m)	131.8 (CH)
4	2.08-2.14 (1H, m)<br>1.90-1.96 (1H, m)	28.6 (CH_2)/28.5 (CH_2)
5	1.54-1.59 (1H, m)<br>1.23-1.28 (1H, m)	32.8 (CH_2)
6<br>7	3.14 (2H, br)	73.23 (CH)/73.18 (CH)
8	1.59-1.64 (1H, m)<br>1.27-1.33 (1H, m)	32.4 (CH_2)
9	2.20-2.25 (1H, m)<br>2.01-2.08 (1H, m)	28.6 (CH_2)/28.5 (CH_2)
10	5.75 (1H, dt, *J* = 15.1, 7.4 Hz)	135.9 (CH)
11	6.04 (1H, dd, *J* = 15.1, 10.4 Hz)	130.6 (CH)
12	6.30 (1H, dt, *J* = 17.0, 10.4 Hz)	137.4 (CH)
13	5.08 (1H, dd, *J* = 17.0, 1.8 Hz)<br>4.94 (1H, dd, *J* = 10.4, 1.2 Hz)	114.9 (CH_2)
6-OH	4.34 (1H, d, *J* = 6.0 Hz)	
7-OH	4.36 (1H, d, *J* = 5.4 Hz)	

[11] Li, B.; Huang, Q.X.; Gao, D.; Liu, D.; Ji, Y.B.; Liu, H.G.; Lin, W.H. New C_{13} lipids from the marine-derived fungus *Trichoderma harzianum*. *J Asian Nat Prod Res* **2015**, 17, 468-474.
